# Ethnic Rhinoplasty: A Middle East-Centered Patient Satisfaction Survey Using the FACE-Q Questionnaire

**DOI:** 10.7759/cureus.40048

**Published:** 2023-06-06

**Authors:** Deoda Maassarani, Raymond Challita, Nancy Zeaiter, Diala Chbib, Joanne Chamy, Imadeddine Farfour, George Ghanime, Ziad Sleiman

**Affiliations:** 1 Plastic and Reconstructive Surgery, Grenoble Alpes University Hospital, Grenoble, FRA; 2 Plastic and Reconstructive Surgery, Faculty of Medicine, Lebanese University, Beirut, LBN; 3 Obstetrics and Gynecology, Faculty of Medicine, Lebanese University, Beirut, LBN; 4 Vascular and Endovascular Surgery, Faculty of Medicine, Lebanese University, Beirut, LBN; 5 Otolaryngology - Head and Neck Surgery, Faculty of Medicine, Lebanese University, Beirut, LBN; 6 Plastic and Reconstructive Surgery, Lebanese Hospital Geitaoui-University Medical Center, Achrafieh, LBN; 7 Plastic and Reconstructive Surgery, Lebanese Hospital Geitaoui-University Medical Center, Ashrafieh, LBN

**Keywords:** ethnic, face-q, satisfaction, esthetic, surgery, plastic, rhinoplasty

## Abstract

Background

Rhinoplasty is a common and complex plastic surgery procedure. The evaluation of surgical success in rhinoplasty is primarily based on patient satisfaction. The purpose of the study is to assess the characteristics of patients who underwent rhinoplasty and their satisfaction using the FACE-Q questionnaire.

Methodology

This was a retrospective, cross-sectional study of patients who underwent primary rhinoplasty, septorhinoplasty, or a revision rhinoplasty from 2010 to 2020 at a single center. Patients were asked to complete the FACE-Q nose score pre and postoperatively. Patients also provided information on their sociodemographic characteristics, smoking status, alcohol consumption, number of rhinoplasty procedures, cause of revision, and respiratory symptoms before rhinoplasty.

Results

This study included 183 patients who underwent rhinoplasty between 2010 and 2020. The mean (SD) age of patients at surgery was 25.92 (8.69) years. There were 156 female respondents (85.2%) and 27 male respondents (14.8%). FACE-Q nose satisfaction scores increased significantly after surgery with a mean of 67.21 ± 22.3 (p = 0.000). The most common reason for revision surgery was tip dissatisfaction.

Conclusions

The findings of this study show that ethnic rhinoplasty, although a complex procedure, can lead to aesthetically pleasing outcomes in a complex population such as the Middle Eastern population.

## Introduction

According to the American Society of Plastic Surgeons, rhinoplasty remains one of the most popular procedures performed for both functional and cosmetic reasons [1]. With over 350,000 nose-reshaping operations performed in 2020, cosmetic rhinoplasty remains the most common plastic surgery performed in the United States [1].

Rhinoplasty is considered a challenging and intricate form of plastic surgery. Despite the skill of the surgeon, the technical obstacles, varied methods, and the challenge of achieving consistent outcomes make it a difficult procedure [2]. Rhinoplasty can be performed to address functional problems and/or esthetic concerns. Cosmetic rhinoplasty aims to improve the appearance of the nose while maintaining nasal functions [2].

In rhinoplasty, patient selection is critical. Despite a good surgical result judged by the surgeon, a significant percentage of patients may be dissatisfied. Several factors, including gender, age, educational level, and, most importantly, patient ethnicity, have been found to play an important role in successful rhinoplasty [3]. In a study conducted by Broer et al. [4], the effects of ethnicity and culture on preferences for nasal shape were examined, and it was found that there were significant variations in aesthetic perception depending on the patient’s gender, age, place of origin, and ethnic background. In the study, computerized images of a model’s nose were manipulated to change the width, root, tip, dorsum, and projection of the lips and chin and were sent to over 13,000 plastic surgeons and individuals from 50 different countries. The comparison of results from participants of Caucasian and East Asian descent revealed the most prominent differences in the projection of the nasal tips, lips, and chins, with East Asian participants preferring more projected nasal tips and less projected lips and chins compared to Caucasian participants [4].

The concept of ethnic rhinoplasty acknowledges the need to take into account variations in both nasal anatomy and cultural views of beauty when performing the surgery. The main distinction between traditional rhinoplasty and ethnic rhinoplasty is the structural differences between Caucasian and non-Caucasian noses. Research has shown that there are racial variations in nasal anatomy. For example, Morgan et al. [5] found that Black individuals have a larger minimum cross-sectional area in the nose compared to both Caucasians and Orientals, and Orientals have a slightly smaller minimum cross-sectional area than Caucasians. Additionally, a previous study conducted by Canbay and Bhatia showed that nasal resistance is lower in Black individuals compared to Caucasians [6].

There are several tools that can be used to evaluate the satisfaction, quality of life, and potential side effects of patients who have undergone rhinoplasty [7]. These tools rely on direct input from the patients themselves, rather than interpretation from a clinician, and provide a perspective on the patient’s experience. This information is particularly important for rhinoplasty, as it allows surgeons to understand and meet the expectations, concerns, and questions of their patients. To this end, plastic surgeons must effectively measure and report patient satisfaction and quality of life after a cosmetic rhinoplasty procedure [7]. Recently, a new instrument called the FACE-Q has been developed by Klassen et al. [8], which has multiple independently functioning scales, many of which are specific to a certain procedure such as rhinoplasty. The FACE-Q assesses various aspects relevant to patients who have undergone facial aesthetic procedures, including appearance, quality of life, and the care process [8].

Recently, the subject of ethnic rhinoplasty has gained significant attention, particularly in international scientific forums. Various ethnic groups exhibit substantial variations in their post-correction expectations and perceptions of their nose appearance [9]. This study aims to evaluate the satisfaction of patients in our country who have undergone rhinoplasty using the FACE-Q questionnaire. The study also aims to identify the factors that affect patient satisfaction. The identification of factors that influence patient satisfaction after rhinoplasty in our country is crucial for several reasons. First, patients from the Middle East, including our country, have unique characteristics such as heavy and thick skin and soft cartilage, which presents specific challenges to the rhinoplasty surgeon [10]. Second, the demographic characteristics of patients undergoing rhinoplasty vary by country, and surgeons must be aware of the impact of these characteristics on patient satisfaction following rhinoplasty [11]. Third, understanding patient expectations, concerns, and questions is critical to achieving successful outcomes in rhinoplasty, and patient-reported outcome instruments can provide data from the patient’s point of view. Finally, no research has been conducted in our country to assess patient satisfaction and outcomes following rhinoplasty, making it essential to conduct studies to fill this gap in knowledge.

## Materials and methods

We conducted a retrospective, cross-sectional study among patients operated on for rhinoplasty at a single center (university hospital) between 2010 and 2020. Institutional review board approval was obtained from the Ethical Committee of Lebanese Hospital Geitaoui in 2021 (approval number: 2021-IRB-001).

Inclusion criteria were patients with no age limitation who had undergone either primary rhinoplasty, septorhinoplasty, or revision rhinoplasty from 2010 to 2020. Exclusion criteria were patients who were followed up for less than one year, those who were not reachable by phone call, those with psychiatric disorders, and those with a history of nasal bone fractures.

Patients who approved our request were asked to give oral informed consent and fill out a questionnaire that included the FACE-Q-Esthetic Satisfaction With Nose Scale, alongside questions to describe our sample, including gender, age, social class (patients with a monthly income of less than one million were classified as lower class, those with a monthly income between one and three million were classified as middle class, and those with a monthly income of more than three million were classified as upper class), address, educational level, smoking/alcohol consumption, the number of cigarettes smoked in a day, the quantity of alcohol consumption per day, the number of rhinoplasties, cause of revision, and respiratory symptoms that they complained about before rhinoplasty.

All rhinoplasty surgeries were performed at the same hospital by two surgeons with at least 10 years of rhinoplasty experience.

All participants were introduced to the rationale of the study before filling out the questionnaire and were informed that participation is voluntary and data recorded anonymously will remain confidential.

Study measurements

The FACE-Q questionnaire (see Appendices) assessed patient satisfaction with their nose appearance and any adverse effects before and after the surgery. The scale is divided into two parts. The first is the Satisfaction With Nose, which includes 10 questions about the patient’s satisfaction with the size, length, width, and appearance of the nose. The second part includes questions about Adverse Effects Regarding the Nose, which includes four questions about any problems or issues experienced before or after the surgery. Patients were asked to rate their level of satisfaction/dissatisfaction on a scale of 1 to 4 (1: very dissatisfied, 2: somewhat dissatisfied, 3: somewhat satisfied, and 4: very satisfied) for the first group of questions and to indicate the extent of any problems or issues on a scale of 1 to 4 (1: not at all, 2: a little, 3: moderately, 4: extremely) for the second group of questions.

The questionnaire was completed preoperatively at the time of the preoperative consultation or one day before the operation. Postoperative answers were obtained from eligible patients orally via phone after sending them an electronic form of the questionnaire. However, because the FACE-Q questionnaire was validated in 2016, all patients admitted to our hospital before the date of validation did not complete the questionnaire preoperatively. This explains the gap in our preoperative data.

The license to use the FACE-Q-Esthetic Satisfaction With Nose Scale and Adverse Effects was obtained from http://www.qportfolio.org/.

Data analysis

Data analysis was done using SPSS version 26 (IBM Corp., Armonk, NY, USA). Descriptive analysis was conducted to describe our sample and obtain measures of the frequency of categorical variables. Regarding the continuous variables, mean and standard deviation (SD) were calculated. Rasch-transformed scores (range = 0-100) were calculated for each patient for the Nose Appearance Scale, and then they were compared from before to after rhinoplasty using the paired-t test. A one-way analysis of variance (ANOVA) test was conducted to assess the association between FACE-Q scores and sociodemographic and clinical factors. Finally, satisfaction and dissatisfaction patterns between male and female patients were compared using the chi-square test. A p-value <0.05 was considered significant.

## Results

Patient characteristics

This study included 183 patients who had undergone rhinoplasty between 2010 and 2020. In total, 101 patients (response rate: 55.1%) responded to the preoperative questionnaire, while all patients (response rate: 100%) responded to the postoperative questionnaire. The mean age (SD) of patients at surgery was 25.92 (8.69) years. The majority of the patients (33.3%) were in the age group of 15-20 years. Overall, 156 (85.2%) of the respondents were female and 27 (14.8%) were males. A description of the study population is shown in Table 1.

**Table 1:** Sociodemographic, lifestyle, and clinical characteristics of the sample. SD: standard deviation

Demographics	
Age at surgery (years), N (%)	
15–20	61 (33.3)
21–25	47 (25.7)
26–30	31 (16.9)
31–35	20 (10.9)
36–39	10 (5.5)
≥40	14 (7.7)
Gender, N (%)	
Male	27 (14.8)
Female	156 (85.2)
Education level, N (%)	
Lower	46 (25.1)
higher	137 (74.9)
Governorates, N (%)	
North Lebanon	9 (4.9)
Mount Lebanon	127 (69.4)
Beirut	36 (19.7)
Bekaa	6 (3.3)
South Lebanon	1 (0.5)
Nabatiye	4 (2.2)
Social class, N (%)	
Lower class (less than 1 million)	32 (17.5%)
Middle class (between 1 and 3 million)	77 (42.1%)
High class (more than 3 million)	74 (40.4%)
Lifestyle characteristics	
Number of cigarettes per day, N (%)	
None	123 (67.2)
Less than 5 cigarettes	33 (18.0)
Between 5 and 20 cigarettes	22 (12.0)
More than 20 cigarettes	5 (2.7)
Alcohol consumption, N (%)	
None	85 (46.4)
Daily alcohol consumption	2 (1.1)
1–2 times/week alcohol consumption	28 (15.3)
1–3 times/month alcohol consumption	38 (20.8)
<1 time/month alcohol consumption	24 (13.1)
3–6 times/week alcohol consumption	6 (3.3)
Clinical characteristics	
Respiratory symptoms before rhinoplasty, N (%)	
No respiratory symptoms	95 (51.9)
One symptom	34 (18.6)
Two or more symptoms	54 (29.5)

Revision rate and causes

In total, 25 of the 183 patients underwent a rhinoplasty revision, representing a revision rate of 13.6%. The most common cause of revision rhinoplasty was aesthetic, with 23 patients requiring surgery for that purpose (92%). Of these, 10 (43.4%) were due to tip dissatisfaction, five (21.7%) due to hump dissatisfaction, and the remaining eight due to other aesthetic issues. However, only two (8.0%) patients required revision for respiratory symptoms, including shortness of breath.

Patient satisfaction and dissatisfaction before and after rhinoplasty

Before the operation, the majority of patients were somewhat dissatisfied or very dissatisfied with all the items of the FACE-Q-Esthetic Satisfaction With Nose questionnaire. Patients’ dissatisfaction was especially high in the items related to the shape of the nose in profile (60.4%) and with how the nose looked in a photo (59.4%). Furthermore, before the operation, no patient was very satisfied with the overall size of the nose, how well the nose suited their face, and how the nose looked from every angle (Table 2).

**Table 2:** Preoperative FACE-Q-Esthetic Satisfaction With Nose checklist.

	Very dissatisfied	Somewhat dissatisfied	Somewhat satisfied	Very satisfied
How satisfied are you with the overall size of your nose?	46 (45.5%)	46 (45.5%)	9 (8.9%)	0 (0.0%)
How satisfied are you with how straight your nose looks?	44 (43.6%)	44 (43.6%)	10 (9.9%)	3 (3.0%)
How satisfied are you with how well your nose suits your face?	43 (42.6%)	46 (45.5%)	12 (11.9%)	0 (0.0%)
How satisfied are you with the length of your nose?	39 (38.6%)	36 (35.6%)	21 (20.8%)	5 (5.0%)
How satisfied are you with the width of your nose at the bottom (from nostril to nostril)?	30 (29.7%)	43 (42.6%)	24 (23.8%)	4 (4.0%)
How satisfied are you with how the bridge of your nose looks (where the glasses sit)?	42 (41.6%)	39 (38.6%)	18 (17.8%)	2 (2.0%)
How satisfied are you with how the tip of your nose looks?	43 (42.6%)	40 (39.6%)	15 (14.9%)	3 (3.0%)
How satisfied are you with the shape of your nose in profile (side view)?	61 (60.4%)	34 (33.7%)	5 (5.0%)	1 (1.0%)
How satisfied are you with how your nose looks in photos?	60 (59.4%)	33 (32.7%)	7 (6.9%)	1 (1.0%)
How satisfied are you with how your nose looks from every angle?	46 (45.5%)	48 (47.5%)	7 (6.9%)	0 (0.0%)

Furthermore, we noticed that before undergoing surgery, few patients were somewhat or very satisfied with the overall size of the nose (8.9%), how straight the nose looked (19.9%), how well the nose suited the face (11.9%), the length of the nose (25.8%), the width of the nose at the bottom (from nostril to nostril) (27.8%), how the bridge of the nose looked (where eyeglasses sit) (19.8%), how the tip of the nose looked (17.9%), the shape of the nose in profile (side view) (6.0%), how the nose looked in photographs (7.9%), and how the nose looked from every angle (6.9%) (Table 2).

On the other hand, after rhinoplasty, the majority of patients reported that they were very satisfied or somewhat satisfied with all the measured outcomes (Table 3).

**Table 3:** Postoperative FACE-Q-Eesthetic Satisfaction With Nose checklist.

	Very dissatisfied	Somewhat dissatisfied	Somewhat satisfied	Very satisfied
How satisfied are you with the overall size of your nose?	7 (3.8%)	18 (9.8%)	78 (42.6%)	80 (43.7%)
How satisfied are you with how straight your nose looks?	14 (7.7%)	22 (12.0%)	67 (36.6%)	80 (43.7%)
How satisfied are you with how well your nose suits your face?	10 (5.5%)	14 (7.7%)	70 (38.3%)	89 (48.6%)
How satisfied are you with the length of your nose?	12 (6.6%)	18 (9.8%)	62 (33.9%)	91 (49.7%)
How satisfied are you with the width of your nose at the bottom (from nostril to nostril)?	13 (7.1%)	25 (13.7%)	62 (33.9%)	83 (45.4%)
How satisfied are you with how the bridge of your nose looks (where the glasses sit)?	14 (7.7%)	24 (13.1%)	53 (29.0%)	92 (50.3%)
How satisfied are you with how the tip of your nose looks?	17 (9.3%)	34 (18.6%)	57 (31.1%)	75 (41.0%)
How satisfied are you with the shape of your nose in profile (side view)?	12 (6.6%)	21 (11.5%)	63 (34.4%)	87 (47.5%)
How satisfied are you with how your nose looks in photos?	14 (7.7%)	31 (16.9%)	66 (36.1%)	72 (39.3%)
How satisfied are you with how your nose looks from every angle?	6 (3.3%)	43 (23.5%)	79 (43.2%)	55 (30.1%)

Patient satisfaction with nose appearance after rhinoplasty

The results of paired t-test showed that the mean score for nose satisfaction after undergoing rhinoplasty (mean = 67.21, SD = 22.3) was significantly greater than that before undergoing rhinoplasty (mean = 26.65, SD = 16.61), indicating a significant improvement in satisfaction with nose post-rhinoplasty (p = 0.000) (Table 4).

**Table 4:** Pre- and post-rhinoplasty comparison of scores. *: p <0.05 is statistically significant; p-value was calculated using the paired t-test. SD: standard deviation; CI: confidence interval

	Pre-rhinoplasty, mean (SD)	Post-rhinoplasty, mean (SD)	Mean difference (95% CI)	P-value
Score	26.65 (16.61)	67.21 (22.3)	40.55 (34.75-46.35)	0.000*

Satisfaction and dissatisfaction patterns between male and female rhinoplasty patients

Before the operation, and for both male and female participants, the majority were somewhat dissatisfied or very dissatisfied with the appearance of the nose. Patient dissatisfaction was especially high in the item related to the shape of the nose in the profile, with more male patients reporting that they were very dissatisfied compared to female patients (70.6% vs. 58.3%) (Table 5).

**Table 5:** Gender differences in each item of the FACE-Q Satisfaction With Nose preoperatively.

	Preoperative		P-value
	Male	Female	
How satisfied are you with the overall size of your nose?			
Very dissatisfied	8 (47.1%)	38 (45.2%)	0.354
Somewhat dissatisfied	9 (52.9%)	37 (44.0%)	
Somewhat satisfied	0 (0.0%)	9 (10.7%)	
Very satisfied	0 (0.0%)	0 (0.0%)	
How satisfied are you with how straight your nose looks?			
Very dissatisfied	6 (35.3%)	38 (45.2)	0.66
Somewhat dissatisfied	10 (58.8%)	34 (40.5%)	
Somewhat satisfied	1 (5.9%)	9 (10.7%)	
Very satisfied	0 (0.0%)	3 (3.6%)	
How satisfied are you with how well your nose suits your face?			
Very dissatisfied	7 (41.2%)	36 (42.9%)	0.64
Somewhat dissatisfied	9 (52.9%)	37 (44.0%)	
Somewhat satisfied	1 (5.9%)	11 (13.1%)	
Very satisfied	0 (0.0%)	0 (0.0%)	
How satisfied are you with the length of your nose?			
Very dissatisfied	7 (41.2%)	32 (38.1%)	0.92
Somewhat dissatisfied	6 (35.3%)	30 (35.7%)	
Somewhat satisfied	4 (23.5%)	17 (20.2%)	
Very satisfied	0 (0.0%)	5 (6.0%)	
How satisfied are you with the width of your nose at the bottom (from nostril to nostril)?			
Very dissatisfied	4 (23.5%)	26 (31.0%)	0.28
Somewhat dissatisfied	11 (64.7%)	32 (38.1%)	
Somewhat satisfied	2 (11.8%)	22 (26.2%)	
Very satisfied	0 (0.0%)	4 (4.0%)	
How satisfied are you with how the bridge of your nose looks (where the glasses sit)?			
Very dissatisfied	7 (41.2%)	35 (41.7%)	0.88
Somewhat dissatisfied	6 (35.3%)	33 (39.3%)	
Somewhat satisfied	4 (23.5%)	14 (16.7%)	
Very satisfied	0 (0.0%)	2 (2.4%)	
How satisfied are you with how the tip of your nose looks?			
Very dissatisfied	7 (41.2%)	36 (42.9%)	0.93
Somewhat dissatisfied	8 (47.1%)	32 (38.1%)	
Somewhat satisfied	2 (11.8%)	13 (15.5%)	
Very satisfied	0 (0.0%)	3 (3.6%)	
How satisfied are you with the shape of your nose in profile (side view)?			
Very dissatisfied	12 (70.6%)	49 (58.3%)	0.72
Somewhat dissatisfied	5 (29.4%)	29 (34.5%)	
Somewhat satisfied	0 (0.0%)	5 (6.0%)	
Very satisfied	0 (0.0%)	1 (1.2%)	
How satisfied are you with how your nose looks in photos?			
Very dissatisfied	9 (52.9%)	51 (60.7%)	0.85
Somewhat dissatisfied	7 (41.2%)	26 (31.0%)	
Somewhat satisfied	1 (5.9%)	6 (7.1%)	
Very satisfied	0 (0.0%)	1 (1.2%)	
How satisfied are you with how your nose looks from every angle?			
Very dissatisfied	5 (29.4%)	41 (48.8%)	0.08
Somewhat dissatisfied	12 (70.6%)	36 (42.9%)	
Somewhat satisfied	0 (0.0%)	7 (8.3%)	
Very satisfied	0 (0.0%)	0 (0.0%)	

On the other hand, more females than males were dissatisfied with how straight the nose looked (45.2% vs. 35.3%), how the nose looked in photos (60.7% vs 52.9%), and how the nose looked in every angle (48.8% vs. 29.4%) (Table 5).

Among satisfied patients, no male patient was very satisfied with all items, while only a few women were fully satisfied with how straight the nose looked (3.6%), the length of the nose (6.0%), the width of the nose at the bottom (4.0%), and how the tip of the nose looked (3.6%) (Table 5).

After the operation, we noticed that males had lower satisfaction when compared to female patients. While 51.9% of female participants were very satisfied with how the bridge of the nose looked, 40.7% of male patients were fully satisfied. Lower satisfaction among male patients was also found in all other items (Table 6).

**Table 6:** Gender differences in each item of the FACE-Q Satisfaction With Nose postoperatively.

	Postoperative		P-value
	Male	Female	
How satisfied are you with the overall size of your nose?			
Very dissatisfied	2 (7.4%)	5 (3.2%)	0.649
Somewhat dissatisfied	3 (11.1%)	15 (9.6%)	
Somewhat satisfied	11 (40.7%)	67 (42.9%)	
Very satisfied	11 (40.7%)	69 (44.2%)	
How satisfied are you with how straight your nose looks?			
Very dissatisfied	3 (11.1%)	11 (7.1%)	0.88
Somewhat dissatisfied	3 (11.1%)	19 (12.2%)	
Somewhat satisfied	10 (37.0%)	57 (36.5%)	
Very satisfied	11 (40.7%)	69 (44.2%)	
How satisfied are you with how well your nose suits your face?			
Very dissatisfied	3 (11.1%)	7 (4.5%)	0.45
Somewhat dissatisfied	1 (3.7%)	13 (8.3%)	
Somewhat satisfied	11 (40.7%)	59 (37.8%)	
Very satisfied	12 (44.4%)	77 (49.4%)	
How satisfied are you with the length of your nose?			
Very dissatisfied	4 (14.8%)	8 (5.1%)	0.26
Somewhat dissatisfied	2 (7.4%)	16 (10.3%)	
Somewhat satisfied	10 (37.0%)	52 (33.3%)	
Very satisfied	11 (40.7%)	80 (51.3%)	
How satisfied are you with the width of your nose at the bottom (from nostril to nostril)?			
Very dissatisfied	3 (11.1%)	10 (6.4%)	0.54
Somewhat dissatisfied	5 (18.5%)	20 (12.8%)	
Somewhat satisfied	9 (33.3%)	53 (34.0%)	
Very satisfied	10 (37.0%)	73 (46.8%)	
How satisfied are you with how the bridge of your nose looks (where the glasses sit)?			
Very dissatisfied	3 (11.1%)	11 (7.1%)	0.56
Somewhat dissatisfied	3 (11.1%)	21 (13.5%)	
Somewhat satisfied	10 (37.0%)	43 (27.6%)	
Very satisfied	11 (40.7%)	81 (51.9%)	
How satisfied are you with how the tip of your nose looks?			
Very dissatisfied	3 (11.1%)	14 (9.0%)	0.98
Somewhat dissatisfied	5 (18.5%)	29 (18.6%)	
Somewhat satisfied	8 (29.6%)	49 (31.4%)	
Very satisfied	11 (40.7%)	64 (41.0%)	
How satisfied are you with the shape of your nose in profile (side view)?			
Very dissatisfied	3 (11.1%)	9 (5.8%)	0.40
Somewhat dissatisfied	1 (3.7%)	20 (12.8%)	
Somewhat satisfied	10 (37.0%)	53 (34.0%)	
Very satisfied	13 (48.1%)	74 (47.4%)	
How satisfied are you with how your nose looks in photos?			
Very dissatisfied	2 (7.4%)	12 (7.7%)	0.75
Somewhat dissatisfied	3 (11.1%)	28 (17.9%)	
Somewhat satisfied	12 (44.4%)	54 (34.5%)	
Very satisfied	10 (37.0%)	62 (39.7%)	
How satisfied are you with how your nose looks from every angle?			
Very dissatisfied	2 (7.4%)	4 (2.6%)	0.10
Somewhat dissatisfied	3 (11.1%)	40 (23.5%)	
Somewhat satisfied	16 (59.3%)	63 (40.4%)	
Very satisfied	6 (22.2%)	49 (31.4%)	

Among dissatisfied patients, male patients also showed higher dissatisfaction rates than female patients, except for the item of how the nose looked from every angle, with about 26% of female patients being very dissatisfied (2.6%) or somewhat dissatisfied (23.5%) compared to 15.5% of dissatisfied male participants (7.4% were very dissatisfied and 11.1% were somewhat dissatisfied) (Table 6).

However, despite these disparities between male and female participants, we did not find a significant difference in satisfaction between them regarding the different items of the questionnaire, whether preoperatively or postoperatively.

Adverse effects

Regarding preoperative and postoperative adverse effects, over half of the patients did not experience any difficulty in breathing (57.4% and 53.0%, respectively), tenderness (82.2% and 65.6%, respectively), swollen or thick-looking skin (82.2% and 74.3%, respectively), or any unnatural bumps or hollows on the nose (77.2% and 74.3%, respectively) (Table 7).

**Table 7:** Adverse effects checklist.

	Not at all		A little		Moderately		Extremely	
	Preoperative N (%)	Postoperative N (%)	Preoperative N (%)	Postoperative N (%)	Preoperative N (%)	Postoperative N (%)	Preoperative N (%)	Postoperative N (%)
Did you experience any difficulty breathing through your nose?	58 (57.4%)	97 (53.0%)	21 (20.8%)	51 (27.9%)	13 (12.9%)	27 (14.8%)	9 (8.9%)	8 (4.4%)
Did you experience any tenderness (e.g., when wearing sunglasses)?	83 (82.2%)	120 (65.6%)	12 (11.9%)	40 (21.9%)	5 (5.0%)	19 (10.4%)	1 (1.0%)	4 (2.2%)
Did you experience swollen or thick-looking skin on the nose?	83 (82.2%)	136 (74.3%)	11 (10.9%)	29 (15.8%)	5 (5.0%)	14 (7.7%)	2 (2.0%)	4 (2.2%)
Did you experience any unnatural appearing bumps or hollows on your nose?	78 (77.2%)	136 (74.3%)	13 (12.9%)	31 (16.9%)	7 (6.9%)	9 (4.9%)	3 (3.0%)	7 (3.8%)

Preoperative adverse effects regarding the nose were moderate difficulty in breathing through the nose (12.9%), tenderness such as when wearing sunglasses (5.0%), the skin of the nose looking thick or swollen (5.0%), and unnatural bumps or hollows on the nose (6.9%). Adverse effects were generally rated more severe after undergoing surgery: moderate difficulty breathing through the nose (14.8%,), tenderness such as when wearing sunglasses (10.4%), and the skin of the nose looking thick or swollen (7.7%). Finally, nine patients reported extreme difficulty in breathing before the surgery, while eight patients cited extreme difficulty breathing through the nose after the operation (Table 7).

Comparison of the FACE-Q satisfaction score with demographic factors, substance use, and clinical variables

A one-way ANOVA was conducted to evaluate the relationship between FACE-Q satisfaction post-rhinoplasty and the different demographic and clinical factors included in this study. Of all the studied variables, we found that only age had significant differences in the FACE-Q Satisfaction With the Nose Scale post-rhinoplasty (p = 0.004) (Table 8).

**Table 8:** Association between demographic, clinical factors, and post-rhinoplasty satisfaction score. SD: standard deviation; df: degree of freedom

Independent variables		N	Mean (SD)	F (df)	P-value
Gender	Male	27	63.33 (23.93)	1.1 (1)	0.288
	Female	156	68.47 (22.97)		
Age (years)	15–20	61	68.56 (21.14)	3.6 (5)	0.004
	21–25	47	73.51 (21.17)		
	26–30	31	62.81 (25.99)		
	31–35	20	55.55 (16.96)		
	36–39	10	85.00 (20.34)		
	≥40	14	60.43 (29.54)		
Education level	Lower	46	69.11 (26.25)	0.2 (1)	0.436
	Higher	137	67.24 (22.05)		
Monthly income (LBP)	Lower class	32	65.34 (25.56)	1.4 (2)	0.228
	Middle class	77	65.26 (24.28)		
	High class	74	71.28 (20.49)		
Smoking status	None	123	68.06 (22.69)	0.61 (3)	0.609
	Less than 5 cigarettes	33	69.09 (23.47)		
	Between 5 and 20 cigarettes	22	66.73 (34.09)		
	More than 20 cigarettes	5	54.40 (30.38)		
Alcohol consumption	None	85	68.55 (23.85)	0.32 (5)	0.897
	1–2 times/week alcohol consumption	28	66.07 (24.49)		
	1–3 times/month alcohol consumption	38	68.32 (21.47)		
	<1 time/month alcohol consumption	24	67.96 (21.28)		
	3–6 times/week alcohol consumption	6	57.00 (31.20)		
Surgeon	Surgeon 1	82	68.33 (24.22)	0.1 (1)	0.745
	Surgeon 2	101	67.21 (22.30)		
Rhinoplasty revision	No	158	69.01 (22.95)	3.7 (1)	0.055
	Yes	25	22.93 (4.58)		
Respiratory symptoms	No symptoms	95	70.37 (22.95)	1.7 (2)	0.178
	One symptom	34	67.71 (25.65)		
	Two and more symptoms	54	63.04 (21.36)		

We noticed that the group of patients aged between 36 and 39 years had the highest FACE-Q scores (85.00 ± 20.34), followed by those aged between 21 and 25 years (73.51 ± 21.17), and those aged between 15 and 20 years (68.56 ± 21.14). However, patients aged between 31 and 35 years had lower scores (55.55 ± 16.96) (Table 9). Post-hoc Bonferroni comparisons showed that the differences between the group of patients aged 31 to 35 years and young adults aged 21 to 25 years were significant (p = 0.045). Likewise, a significant difference was found between the group of patients aged 31 to 35 years and those aged 36 to 39 years (p = 0.012) (Table 9).

**Table 9:** Post-hoc multiple comparisons: post-rhinoplasty FACE-Q Satisfaction With Nose score using Bonferroni. *: The mean difference is significant at the 0.05 level.

Age categories (years)	(J) Age categories	Mean difference (I-J)	Standard error	Significance	95% confidence interval	
					Lower bound	Upper bound
15–20	21–25	-4.953	4.334	1.000	-17.85	7.94
	26–30	5.751	4.925	1.000	-8.90	20.41
	31–35	13.007	5.754	0.375	-4.11	30.13
	36–39	-16.443	7.618	0.484	-39.11	6.23
	≥40	8.129	6.617	1.000	-11.56	27.82
21–25	15–20	4.953	4.334	1.000	-7.94	17.85
	26–30	10.704	5.166	0.596	-4.67	26.08
	31–35	17.961^*^	5.961	0.045	0.22	35.70
	36–39	-11.489	7.776	1.000	-34.63	11.65
	≥40	13.082	6.799	0.839	-7.15	33.31
26–30	15–20	-5.751	4.925	1.000	-20.41	8.90
	21–25	-10.704	5.166	0.596	-26.08	4.67
	31–35	7.256	6.404	1.000	-11.80	26.31
	36–39	-22.194	8.121	0.104	-46.36	1.97
	≥40	2.378	7.190	1.000	-19.02	23.77
31–35	15–20	-13.007	5.754	0.375	-30.13	4.11
	21–25	-17.961^*^	5.961	0.045	-35.70	-0.22
	26–30	-7.256	6.404	1.000	-26.31	11.80
	36–39	-29.450^*^	8.648	0.012	-55.18	-3.72
	≥40	-4.879	7.781	1.000	-28.03	18.27
36–39	15–20	16.443	7.618	0.484	-6.23	39.11
	21–25	11.489	7.776	1.000	-11.65	34.63
	26–30	22.194	8.121	0.104	-1.97	46.36
	31–35	29.450^*^	8.648	0.012	3.72	55.18
	≥40	24.571	9.245	0.129	-2.94	52.08
≥40	15–20	-8.129	6.617	1.000	-27.82	11.56
	21–25	-13.082	6.799	0.839	-33.31	7.15
	26–30	-2.378	7.190	1.000	-23.77	19.02
	31–35	4.879	7.781	1.000	-18.27	28.03
	36–39	-24.571	9.245	0.129	-52.08	2.94

In terms of gender, we found that female participants had higher postoperative FACE-Q scores compared to male participants (68.47 ± 22.97 vs. 63.33 ± 23.93, p = 0.288). Regarding education, those with lower education levels had slightly higher satisfaction scores postoperatively compared to those with higher education levels (69.11 ± 26.25 vs. 67.24 ± 22.05, p = 0.436). Furthermore, patients from the upper class had higher postoperative satisfaction scores (71.2820.49) than those from the middle and lower classes (65.2624.28 and 65.3425.56, respectively, p = 0.228). Regarding smoking status and alcohol consumption, the highest mean postoperative satisfaction scores were found among patients who smoked 11 to 15 cigarettes per day (69.09 ± 23.47, p = 0.609) and those who reported that they did not drink alcohol (68.55 ± 23.85, p = 0.897). Almost similar postoperative FACE-Q scores were found among patients operated on by two different surgeons (68.33 ± 24.22 vs. 67.21 ± 22.30, p = 0.745). Furthermore, patients who underwent primary rhinoplasty were found to have higher satisfaction scores post-rhinoplasty in comparison with those who had undergone a rhinoplasty revision. Finally, we found that the mean FACE-Q satisfaction scores post-rhinoplasty were the highest for patients with no respiratory symptoms score (70.37 ± 22.95) compared to those with one symptom (67.71 ± 25.65), as well as those with two symptoms and more (67.71 ± 25.65, p = 0.178).

## Discussion

The procedure of rhinoplasty is the most commonly performed facial cosmetic surgery globally and is considered one of the toughest and most complicated surgeries [2,12]. To achieve successful results in rhinoplasty, it is crucial for the surgeon to understand the expectations and worries of the patient. Measuring patient satisfaction after surgery is difficult as there is no standard method for it [13]. However, FACE-Q scales have been developed to measure patient satisfaction with facial aesthetics by evaluating over 40 different scales and checklists, including the patient’s facial appearance, quality of life, and any negative effects [8]. This study was organized to study preoperative and postoperative patient satisfaction at least one year post-rhinoplasty in a Middle Eastern ethnic group and to study the demographic characteristics of this subgroup.

This study found a significant improvement in nose satisfaction after rhinoplasty (p = 0.000), which is consistent with other studies. Schwitzer et al. [7] were the first to use the FACE-Q scale to examine the changes in patient satisfaction with their facial and nasal appearance, as well as the quality of life, after undergoing rhinoplasty. The study found that there was a significant increase in satisfaction scores related to various aspects of the nose, such as size, shape, profile, appearance in the mirror, and photographs [7]. The creators of the FACE-Q rhinoplasty scale also conducted a comparison study of 23 patients before and after the surgery, which showed an improvement in the score for the Satisfaction with Nose and Nostrils scale [14].

Generally, patients who undergo rhinoplasty are less satisfied with their postoperative appearance compared to those who receive other cosmetic procedures [15]. This could be because rhinoplasty is considered a complex surgical procedure. Unrealistic patient expectations may also play a role [2]. To address this, it is recommended that rhinoplasty patients be interviewed twice before the surgery to understand their reasons for seeking the procedure and ensure their expectations are realistic. This also allows for an explanation of what improvements can be made and how [2]. For example, patients who initially sought improvement for functional reasons may express more concerns about the appearance of their nose during the interview, and after the surgery, they place greater importance on the aesthetic outcome than their breathing ability [2].

In terms of postoperative complications, 47% of patients reported little, moderate, or extreme difficulty breathing through the nose. These findings were lower than those reported in the study by Kalaaji et al. [16] which included 214 Norwegian patients and found that more than 60% of patients reported difficulty breathing through the nose postoperatively. One possible reason for this outcome could be that temporary swelling is a common result of surgery and can lead to temporary obstruction of the nasal airway.

As mentioned previously, the nose esthetic is perceived differently among different ethnicities and cultures. Some studies have shown that people in Asia prefer a more obtuse angle in the nose, a rounder tip, and a smaller projection, while African American women prefer maintaining balance and harmony in the nose, with a straighter dorsum, more defined tip, and slight alar flaring. In the Gulf region, both men and women dislike a retrousse dorsum and prefer a straight or slightly higher profile. However, people from Lebanon, Syria, Turkey, Egypt, and Morocco typically prefer more tip projection.

The importance of considering ethnicity and cultural factors when performing rhinoplasty is highlighted here. The goal of ethnic rhinoplasty is to avoid drastically changing a person’s ethnic identity or appearance, and plastic surgeons should take these factors into account when determining the surgical approach. A study by Schwitzer et al. found that non-Caucasian women were less likely to show significant improvements in satisfaction with their noses following the surgery, possibly due to different expectations. This suggests that surgeons should approach ethnic rhinoplasty differently for non-Caucasian patients.

The success of ethnic rhinoplasty depends on preserving the patient’s ethnic identity and appearance [10]. It is crucial for a plastic surgeon to take into account the patient’s ethnicity and cultural background when performing rhinoplasty, as these factors impact the patient’s desires and surgical approach [17]. In a study by Schwitzer et al. [18], 67.8% of American women included were Caucasian, while 32.2% were non-Caucasian. The results showed that Caucasian women experienced a statistically significant improvement in satisfaction with facial appearance and quality of life, whereas non-Caucasians did not. Therefore, non-Caucasians were less likely to have a significant improvement in satisfaction with the appearance of their nose after rhinoplasty compared to Caucasians. The authors suggest that non-Caucasians may have different expectations, highlighting the importance of different approaches for each patient [18].

In this study, we found that our participants had a particularly high level of dissatisfaction with the shape of their nose in profile (60.4%) and how it appears in photographs (59.4%) before undergoing rhinoplasty. This aligns with the findings of the study by Kalaaji et al. [16] among the Norwegian population, in which 79.6% and 75.5% of participants expressed significant dissatisfaction with the shape of their nose in profile view and how it appears in photographs, respectively. This high level of dissatisfaction with the shape of the nose in profile and in photographs among patients in our study may be attributed to cultural and societal factors. In many cultures in the Middle East, a strong emphasis is placed on physical appearance, and the nose is often considered a defining feature of one’s appearance. Additionally, traditional beauty standards in the Middle East may also place a greater emphasis on the shape of the nose in profile and in photographs.

The finding that similar levels of dissatisfaction with the shape of the nose in profile and in photographs was reported in a study conducted in Norway and one conducted in Lebanon may suggest that concerns about the appearance of the nose are not limited to a specific cultural or ethnic group. It is possible that there are universal aesthetic standards for the appearance of the nose that are commonly held across different cultures and populations. Additionally, it could be that social media and the widespread access to images of people from all around the world have increased exposure to different facial features and have led to the development of similar aesthetic preferences. Another possibility is that there may be cultural and societal factors in our country that contribute to a heightened focus on the appearance of the nose. It could be that the culture places a high value on physical appearance, and this emphasis on appearance leads to a higher level of dissatisfaction with the shape of the nose. Additionally, it could be that there are specific cultural or societal pressures in our country that lead people to be more self-conscious about the appearance of their noses.

Previous research has found that several factors influence patient satisfaction, including gender, age, education level, substance use, and patient’s level of expectation [3]. In this study, we attempted to investigate the relationship between patients’ age, gender, educational level, and monthly income with patients’ satisfaction using the FACE-Q Satisfaction With Nose score. Unfortunately, of all studied variables, only age was found to be significantly associated with patient satisfaction.

Age is considered to be a significant factor that has been shown to play an important role in determining the patient satisfaction score. Our findings show that patients under the age of 40 had higher postoperative FACE-Q scores than those over the age of 40. These findings contradict those found in the literature. Several studies have revealed that younger patients are less satisfied than older patients [17,19,20]. These findings are explained by the fact that younger patients have higher expectations and have difficulty accepting changes in their own image [17,21]. For instance, Arima et al. [21] in a study to determine the effect of patient age on satisfaction reported that satisfaction levels were lower in patients under 30 years old than those over 30 years old. Based on these findings, the authors proposed that younger patients undergoing rhinoplasty require more detailed preoperative guidance, as well as complete information on the surgery’s limitations, to achieve a satisfactory outcome [21].

Our study revealed that females had slightly higher satisfaction scores compared to males. This is in line with the findings of Khansa et al. [15] who conducted a study to compare satisfaction levels between male and female rhinoplasty patients and reported higher satisfaction rates among females [15]. Additionally, Slator et al. discovered that male rhinoplasty patients were more likely to experience depression and dissatisfaction compared to females. Another study on male rhinoplasties concluded that male patients often had non-specific complaints and a limited understanding of their deformity [22]. One explanation for the higher satisfaction rates among women may be that women are more likely to express their emotions and communicate their satisfaction to their surgeon, leading to higher reported satisfaction scores.

In this study, the prevalence of revision rhinoplasties was 13.6%, and this finding was consistent with that reported in the literature where revision rates varied between 5% and 15% [23-25]. A Saudi Arabian study conducted among 1,370 rhinoplasty patients to investigate the prevalence of revision rhinoplasty, its main function and cosmetic causes, and the possible associated factors found a revision prevalence of 8%. Patients seeking revision rhinoplasty had numerous functional and aesthetic concerns. In our study, the most common cause of revision rhinoplasty was aesthetic (92%), and only 8% of patients required revision for functional concerns. Prior studies have demonstrated similar results, where cosmetic complaints were more common than functional complaints among revision rhinoplasty patients.

Finally, our study found that patients who underwent revision rhinoplasty were less satisfied with their postoperative results. This may be due to the psychological impact of previous failed rhinoplasty procedures, and therefore, may have higher expectations or specific goals for the revision procedure, making it less likely for these patients to be satisfied with the results [17]. Another factor could be that revision surgeries are often more complex and involve more tissue manipulation, which increases the risk of complications and may prolong the recovery time, and may not meet the desired outcomes.

Our study has a few limitations. It is a retrospective, descriptive, uni-centric study with a relatively small sample. Adding to this, our gap in data wherein a certain number of patients failed to respond to the FACE-Q questionnaire, and all patients operated on before 2016 who did not possess a preoperative FACE-Q score in their medical records.

## Conclusions

We conducted a study to specify the nasal ethnic and demographic characteristics of our patients undergoing rhinoplasty. The majority of the patients were satisfied with the aesthetic outcome. Thus, individualized rhinoplasty, although a complex procedure, can lead to aesthetically pleasing outcomes in a complex population such as the Middle Eastern population. However, larger, prospective, multicenter, randomized studies are needed to further evaluate ethnic rhinoplasty in diverse populations.

## Appendices

**Table 10:** FACE-QTM - Satisfaction With Nose. Copyright 2013 Memorial Sloan Kettering Cancer Center, New York, USA. All rights reserved.

	Very dissatisfied	Somewhat dissatisfied	Somewhat satisfied	Very satisfied
a. The width of your nose at the bottom (from nostril to nostril)?	1	2	3	4
b. The length of your nose?	1	2	3	4
c. How the bridge of your nose looks (where glasses sit)?	1	2	3	4
d. How well your nose suits your face?	1	2	3	4
e. How straight your nose looks?	1	2	3	4
f. The overall size of your nose?	1	2	3	4
g. The shape of your nose in profile (side view)?	1	2	3	4
h. How your nose looks in photos?	1	2	3	4
i. How the tip of your nose looks?	1	2	3	4
j. How your nose looks from every angle?	1	2	3	4

**Table 11:** FACE-QTM - Adverse Effects: Nose. Copyright 2013 Memorial Sloan-Kettering Cancer Center, New York, USA. All rights reserved.

	Not at all	A little	Moderately	Extremely
a. Did you experience any difficulty in breathing through your nose?	1	2	3	4
b. Did you experience any tenderness (e.g., when wearing sunglasses)?	1	2	3	4
c. Did you experience a swollen or thick-looking skin of the nose?	1	2	3	4
d. Did you experience any unnatural appearing bumps or hollows on your nose?	1	2	3	4

**Table 12:** FACE-QTM - Satisfaction With Nose Conversion Table. Instructions: Higher scores reflect a better outcome. If missing data is less than 50% of the scale’s items, insert the mean of the completed items. Use the Conversion Table below to convert the raw scale summed score into a score from 0 (worst) to 100 (best). Copyright 2013 Memorial Sloan-Kettering Cancer Center, New York, USA. All rights reserved.

Sum score	Equivalent Rasch transformed score (0–100)
10	0
11	15
12	20
13	24
14	28
15	30
16	33
17	35
18	37
19	39
20	40
21	42
22	44
23	45
24	47
25	49
26	50
27	52
28	54
29	56
30	58
31	60
32	62
33	65
34	67
35	70
36	74
37	78
38	83
39	90
40	100
